# Identification of pathological RA endotypes using blood-based biomarkers reflecting tissue metabolism. A retrospective and explorative analysis of two phase III RA studies

**DOI:** 10.1371/journal.pone.0219980

**Published:** 2019-07-24

**Authors:** J. P. M. Blair, C. Bager, A. Platt, M. Karsdal, A. -C. Bay-Jensen

**Affiliations:** 1 ProScion, Herlev, Denmark; 2 University of Copenhagen, Faculty of Health and Medical Sciences, Copenhagen, Denmark; 3 Target & Translational Science, Respiratory, Inflammation and Autoimmunity (RIA), IMED Biotech Unit, AstraZeneca, Gothenburg, Sweden; 4 Rheumatology, Nordic Bioscience, Biomarkers and Research, Herlev, Denmark; SERGAS and IDIS, SPAIN

## Abstract

There is an increasing demand for accurate endotyping of patients according to their pathogenesis to allow more targeted treatment. We explore a combination of blood-based joint tissue metabolites (neoepitopes) to enable patient clustering through distinct disease profiles. We analysed data from two RA studies (LITHE (N = 574, follow-up 24 and 52 weeks), OSKIRA-1 (N = 131, follow-up 24 weeks)). Two osteoarthritis (OA) studies (SMC01 (N = 447), SMC02 (N = 81)) were included as non-RA comparators. Specific tissue-derived neoepitopes measured at baseline, included: C2M (cartilage degradation); CTX-I and PINP (bone turnover); C1M and C3M (interstitial matrix degradation); CRPM (CRP metabolite) and VICM (macrophage activity). Clustering was performed to identify putative endotypes. We identified five clusters (A-E). Clusters A and B were characterized by generally higher levels of biomarkers than other clusters, except VICM which was significantly higher in cluster B than in cluster A (p<0.001). Biomarker levels in Cluster C were all close to the median, whilst Cluster D was characterised by low levels of all biomarkers. Cluster E also had low levels of most biomarkers, but with significantly higher levels of CTX-I compared to cluster D. There was a significant difference in ΔSHP score observed at 52 weeks (p<0.05). We describe putative RA endotypes based on biomarkers reflecting joint tissue metabolism. These endotypes differ in their underlining pathogenesis, and may in the future have utility for patient treatment selection.

## Introduction

Rheumatoid Arthritis (RA) is a heterogeneous chronic inflammatory and autoimmune disease, characterised by pain and swelling of joints, progressive joint deterioration, bone erosions and impaired function, often leading to disability[1]. Due to the heterogeneity and the complex aetiology of the disease, rates of drug response in RA patients varies from compound to compound. A systematic review of effectiveness of anti-TNF compounds found the mean percentage of ACR-20 responders was 60.8% and EULAR responders 70.5%, leaving a large proportion still unresponsive[2]. Development and selection of treatment is therefore a challenging process making it difficult to deliver targeted treatments.

Currently treatment is based on the use of disease activity markers such as disease activity scores (e.g. DAS28), radiographic scores and other clinical assessments to gauge a patient’s state, progress and response to treatment. This does not take into account more intricate pathological differences between the patients but rather more systemic levels of inflammation. By adopting a precision medicine approach, one aims to improve response rates through the use of biomarkers facilitating the identification of patients most likely to benefit[3].

RA may comprise of multiple clinical and molecular endotypes[4], being defined as subtypes of a disease with distinctive underlying pathological mechanisms[4,5]. A patient’s clinical presentation, response to treatment and rates of disease progression may be determined by a patient’s endotype. Thus, well characterised endotypes may provide patients and physicians with a means for targeted treatment against a patient’s individual molecular disease pathogenesis. Moreover, endotypes may provide insight into novel pathways and thereby discovery of new drug mode[4].

In order to begin designing such a tool to identify distinct endotypes in RA, it is vital to have an understanding of underlying pathological mechanisms. Type I, II and III collagens are the most prevalent structural proteins in the joint, and among the most prevalent in the body[6]. Various clinical and preclinical studies have shown in increase in type I, II and III collagen degradation in RA due to an increase in proteolytic activity[7,8]. Multiple biomarkers have been developed that measure such tissue metabolites in blood of patients with arthritis[9]. Examples of such tissue metabolites are: i) CTX-I and PINP, which are measures of bone resorption and formation;[8,10] ii) C1M and C3M, which reflect MMP mediated degradation of collagen 1 and 3 respectively;[11,12] iii) C2M, measuring MMP mediated collagen 2 degradation;[13] iv) CRPM, CRP degradation;[14] and v) VICM, MMP driven citrullinated vimentin degradation[15]. These are also examples of tissue metabolites which have been shown to elevated in RA, associated with disease activity[16] and treatment response[9]. As these metabolites are a reflection of tissue associated disease mechanism, they could be useful tools to identify different endotypes in RA.

The aim of this study was to demonstrate how different putative endotypes representing underlying pathologies of RA, can be identified through the use of joint specific serological biomarkers.

## Materials and methods

### Studies

**Fig 1:**
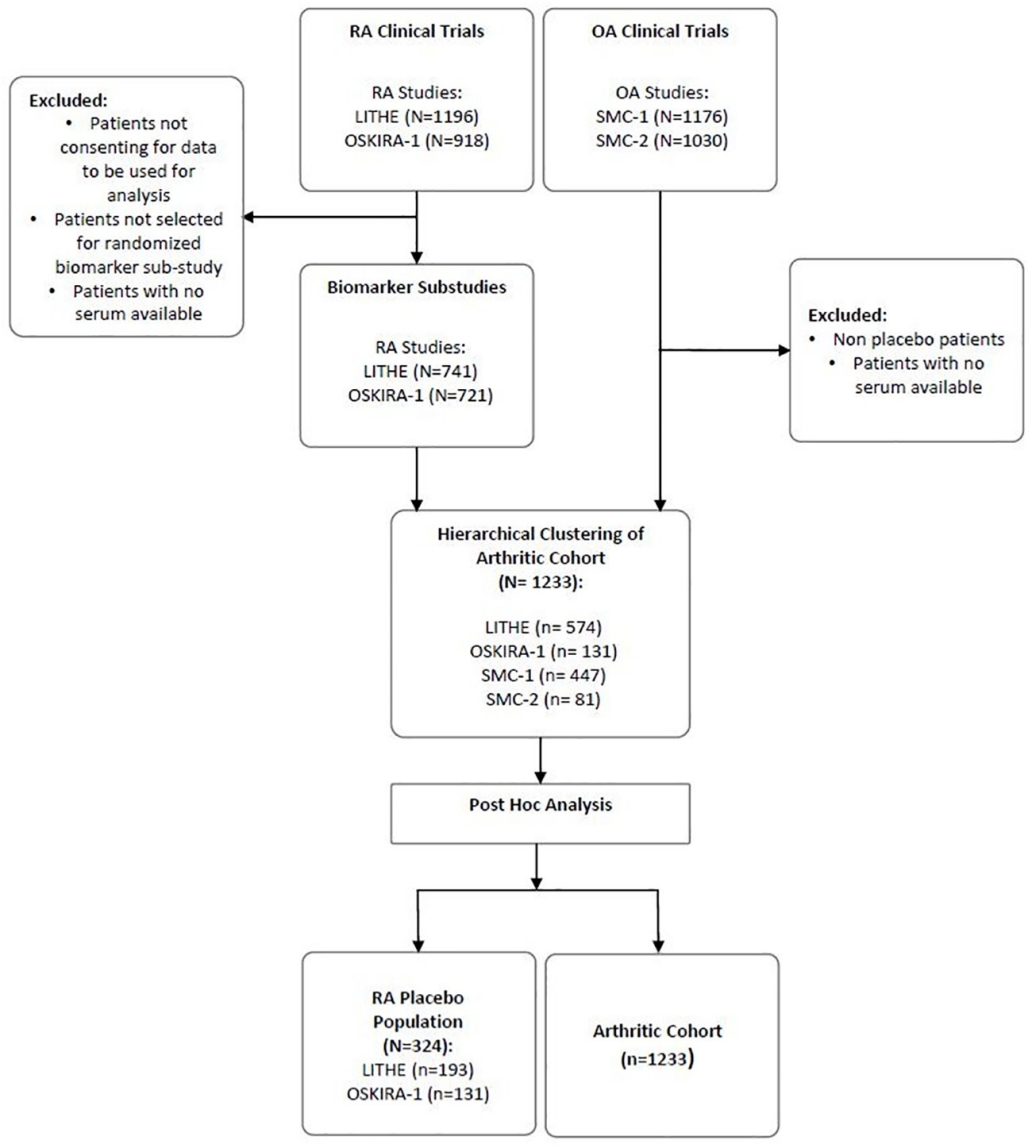
Four biomarker sub-studies of clinical trials were pooled for clustering before further analysis of each cluster. A random selection of patients from the RA clinical trials were selected for biomarker analysis. Patients with missing data from the panel of biomarkers analysed were removed from the study when included in the arthritic cohort.

Four cohorts were pooled for this study, including two RA studies; LITHE (N = 1196, NCT00106535) and OSKIRA-1 (N = 918, NCT01197521) and two OA studies; SMC01 (N = 1176, NCT00486434) and SMC02 (N = 1030, NCT00704847) all of which have been described in detail before[17–20]. The RA studies included patients with moderate to severe disease and with a high disease activity. Patients from the OA studies were included in order to enrich the cohort with a disease population with low inflammation but with known joint disease as characterised by X-ray and by symptomatic scores, providing a broader range of biomarker levels. Patients were randomly selected from the clinical trials to participate in biomarker sub-studies of all phase III clinical trials for RA (LITHE and OSKIRA-1) and placebo arms of OA studies (SMC01 and SMC02) (Fig 1). Patients in LITHE all had active moderate to severe RA with inadequate response to methotrexate (MTX). OSKIRA-1 had similar inclusion criteria, selecting patients with active RA and inadequate response to MTX. Both SMC trials recruited patients with a medical history and symptoms of knee OA. Further details of the patient demographics from each study can be read in Table 1. Patients from any cohort with missing biomarker measurements at baseline were excluded from the study. No ethical approval was required as no patient recruitment or data collection was carried out for this study. The authors did not have access to any identifying participant information.

**Table 1:** Baseline patient demographics for four cohorts used in this study, LITHE, OSKIRA, SMC1 and SMC2. All measurements were taken at the start of each of the clinical trials prior to treatment.

	LITHE	OSKIRA	SMC1	SMC2	p-value
**Cohort characteristics**					
**Disease Indication**	RA	RA	OA	OA	-
**N**	574	131	447	81	-
**Age (± SD)**	52.1 ± 12.3	*	65.7 ± 6.1	64.4 ± 6.38	< 0.001
**% Female**	82.8	83.2	65.4	63.5	0.26
**BMI (mean ± SD) (n)**	27.4 ± 6.0 (567)	27.62 ± 5.3 (131)	28.4 ± 4.9 (447)	28.5 ± 4.4 (81)	0.002
**Burden of disease activity**					-
**DAS28**	6.5 ± 0.92 (563)	5.7 ± 0.81 (73)	NA	NA	
**Pain**					
**VAS pain**	54.4 ± 22.1 (568)				
**HAQ pain**		61.3 ± 19.7 (130)			
**WOMAC pain**			45.2 ± 19.3 (447)	50.6 ± 20.5 (81)	
**X-ray scores**					
**Erosion scores**	16.8 ± 16.0 (549)	21.9 ± 33.7 (131)			
**Kellgren Lawrence = 2**			87.0%	76.5%	
**Kellgren Lawrence = 3**			13.0%	23.5%	

* Ages were reported in ranges for OSKIRA: <25 (0.7%), 25–34 (6.8%), 35–44 (13.7%), 45–54 (28.1%), 55–64 (32.8%), 65–74 (16.9%) and 75–84 (0.7%)

Endpoints in each RA study were measured according to ACR and EULAR criteria[21], and include radiographic measurements, DAS-28 disease activity scores as well as patient and physician reported outcomes. Erosion (ERN), joint space narrowing (JSN) and modified sharp score (SHP) were calculated at baseline and 24 weeks for LITHE and OSKIRA, and at 52 weeks for LITHE. Delta (Δ) ERN, JSN and SHP were calculated by subtracting baseline score from follow-up scores at week 24 and 52 where available for the placebo patients, to remove treatment bias.

### Biomarker measurements

Several serological biomarkers were measured in each cohort and selected due to the specific tissue metabolite they represented. A total of 20 biomarkers were measured across all trials, with 7 biomarkers common to all studies and analysed post hoc in this paper. All samples were destroyed after measurement according protocol. These included: C2M (cartilage degradation)[22,23]; CTX-I[24] and PINP (bone resorption and formation)[13,25]; C1M and C3M (interstitial matrix degradation)[9,11,12,26]; CRPM (CRP metabolite)[14,23], and VICM (macrophage activity)[16,27]. The biomarkers were measured in serum samples following the manufactures protocols and under Good Laboratory Practice (GLP).

All variables were calibrated according to the assay they had been measured in to control for possible batch effect. Each biomarker was then log transformed and min-max normalised to allow for direct comparison of each of the variables.

### Clustering protocol

Three statistical heuristics were evaluated in order to identify cluster tendency and the number of inherent clusters in the data. The GAP statistic[28], silhouette method[29] and elbow method[30] were all used to assess this, with the majority rule used to indicate number of clusters.

Patient clustering was then performed using Ward hierarchical clustering. The statistical significance of the resulting cluster separations was assessed using permutation testing as described by Serviss et al.[31] using 100 iterations. ANOVA and Chi squared tests were used to identify differences of clinical measures between the placebo patients in each of the clusters. In order to identify individual differences between clusters, Tukey ad hoc test was used.

All data handling and statistical analysis was performed using R software (version 3.4.1, R Development Core Team, 2012), with use of *factoextra* (version 1.0.5) and *pheatmap* (version 1.0.8). Permutation testing was performed using the *ClusterSignificance* package (version 1.4.1). Statistics were computed using the *stats* package (version 3.4.1).

## Results

### Study overview

Patients with serological markers measured at baseline (BL) were selected from study populations (N = 1233, Table 1). Patients from the OA studies (SMC01 and SMC02) were significantly older and had significantly higher BMI than patients in the RA cohorts (LITHE and OSKIRA) (Age, p < 0.001; BMI, p = 0.002). There was no significant difference in the percentage of males in the OA cohorts.

### Description of clusters

**Fig 2:**
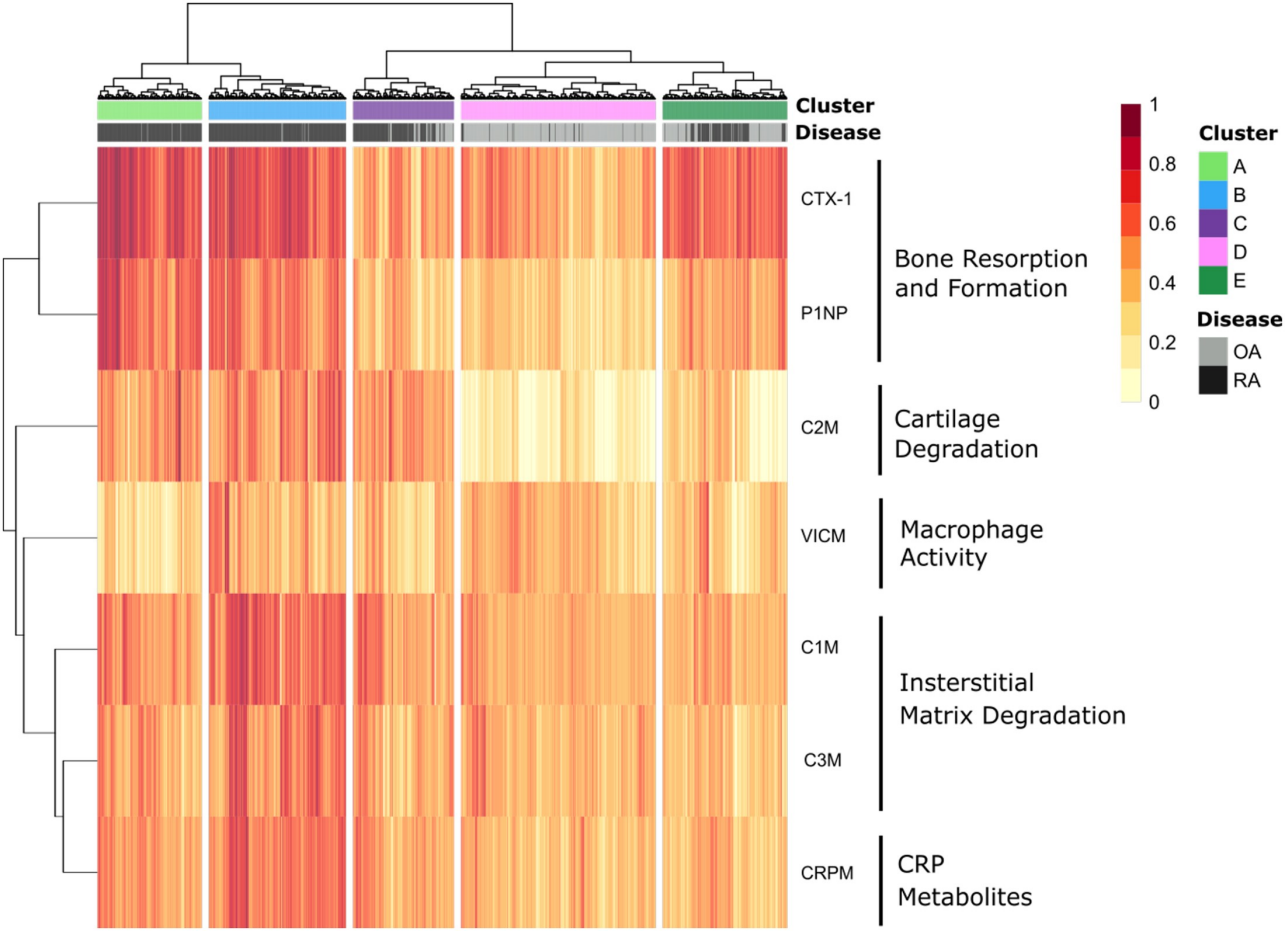
1233 patients from 4 clinical trials; two rheumatoid arthritis (RA) and two osteoarthritis (OA), were clusters based on 7 tissue specific blood based biomarkers measuring bone resorption and formation, cartilage degradation, macrophage activity, interstitial matrix degradation and CRP metabolites. 5 clusters were found with two high inflammatory RA endotypes and one low inflammatory RA endotype, and a final “mixed” endotype. OA patients were clearly separated from RA patients as expected.

Hierarchical clustering was performed based on a panel of 7 tissue specific serological biomarkers measuring bone resorption and formation (CTX-1 and P1NP), cartilage degradation (C2M), macrophage activity (VICM), interstitial matrix degradation (C1M and C3M), and CRP metabolites (CRPM). This analysis revealed 5 distinct clusters from based on patient biomarker profiles, which can be seen in the heatmap in Fig 2. Permutation testing revealed all clusters were statistically significantly different from each other, all with p < 0.01.

Clusters A (194 subjects) and B (256 subjects) were comprised of the majority of the RA patients (97.9% and 98% RA patients respectively). Cluster C (188 subjects), D (363 subjects) and E (232 subjects) contained lower percentages of RA patients (74.5%, 5.8% and 44.3% respectively). This was reflected in the demographics and biomarker profiles of the patients. A full description of cluster demographics and distribution can be found in Table 2.

**Table 2:** Descriptive statistics for each cluster identified through hierarchical clustering, with p-values calculated using ANOVA test to identify differences between the clusters.

Clusters	A	B	C	D	E	p-value
**N**	194	256	188	363	232	-
**RA/OA %**						
**% of OA Cohort**	<1	9.4	9.4	65.0	24.4	
**% of RA Cohort**	27.0	35.6	19.9	2.9	15.0	
Age mean*	51.9 ± 12.7	51.4 ± 12.6	55.8 ± 11.1	64.1 ± 6.8	62.7 ± 10.1	<0.005
**% Female**	80.2	79.7	84.1	61.8	80.0	<0.005
**BMI mean (SD)**	27.4 ± 5.2 (191)	27.5 ± 6.4 (256)	28.0 ± 6.1 (184)	27.9 ± 4.5 (363)	27.1 ± 4.5 (232)	<0.005
**% RA**	97.9	98.0	74.5	5.8	44.3	-

*OSKIRA was omitted due to data being reported in ranges. P-value calculated using ANOVA or chi squared test.

Cluster A and B, which were predominantly RA clusters, had higher levels of C2M, CRPM, C1M and C3M compared to clusters C, D and E. This is indicative of high levels of cartilage turnover, CRP metabolites and interstitial matrix turnover respectively, typically observed in RA patients[32,33]. Both bone markers (P1NP and CTX-I) were also significantly elevated in clusters A and B in comparison to clusters C, D and E (p<0.001). Both Clusters A and B were characterised by consistently high levels of all biomarker measures, indicating cluster A and B appear to reflect endotypes of high disease activity. The difference between cluster A and B was the level of VICM (macrophage activity) that was significantly lower in cluster A compared to cluster B (p<0.001).

Cluster C had a mix of RA and OA patients and did not appear to have a particularly defining characteristic, all biomarker levels being close to the median. Cluster D, the largest cluster and predominantly an OA cluster, had relatively moderate levels of tissue turnover, characterising patients with lower disease activity as expected of OA patients. Furthermore, cluster D was characterised by significantly low levels of C2M, indicating lower cartilage turnover than patients from clusters A, B and C (p < 0.01). Cluster D therefore reflects an endotype with low bone activity, low inflammation and low cartilage activity. While cluster E, a mixed RA and OA cluster, shared many of the characteristics of cluster D, including low levels of all inflammatory markers, and lower levels of cartilage turnover, a notable exception was the higher levels of bone turnover biomarkers observed. Thus, cluster E appears to reflect an endotype with high bone activity and low inflammation.

### Association between clusters and RA clinical assessments in placebo patients

We looked at the RA placebo patients across the 5 clusters in order to identify differences in clinically relevant variables (Table 3).

**Table 3:** Mean and standard deviation of clinical variables for placebo patients in each cluster identified, where p-values have been measured using ANOVA test or Chi squared.

Cluster	A	B	C	D	E	p-value
**N RA**	81	113	54	16	60	-
**Age**	51.2 ± 13.2 (58)	50.7 ± 13.3 (81)	52.9 ± 10.8 (34)	56 ± 10.3 (4)	56.9 ± 10.4 (16)	0.40
**BMI**	27.1 ± 4.7 (80)	28.7 ± 7.6 (113)	27.6 ± 6.5 (53)	27.8 ± 4.01 (16)	26.8 ± 5.32 (60)	0.32
**Disease duration**	9.8 ± 7.7 (81)	9.3 ± 8.4 (113)	9.15 ± 7.8 (54)	10.03 ± 7.91 (16)	8.8 ± 8.96 (60)	0.96
**No. Prev. DMARDS**	1.6 ± 1.3 (58)	1.8 ± 1.5 (81)	1.9 ± 2.1 (34)	1 ± 0.82 (4)	1.6 ± 1.8 (16)	0.83
**% Previous DMARDs**	63.0 (81)	61.1 (113)	61.1 (54)	43.8 (16)	45 (60)	0.139
**HAQ**						
**BL**	1.5 ± 0.64 (73)	1.7 ± 0.59 (107)	1.6 ± 0.64 (53)	1.5 ± 0.61 (16)	1.6 ± 0.52 (57)	0.53
**Δ24**	-0.32 ± 0.61 (51)	-0.43 ± 0.5 (68)	-0.25 ± 0.66 (40)	-0.57 ± 0.47 (15)	-0.23 ± 0.4 (52)	0.08
**Δ52**	-0.24 ± 0.55 (25)	-0.44 ± 0.64 (29)	-0.62 ± 0.37 (12)	0.31 ± 0.27 (2)	-0.25 ± 0.47 (6)	0.12
**DAS**						
**BL**	6.3 ± 0.91 (80)	6.6 ± 0.95 (111)	6.1 ± 1.0 (53)	5.9 ± 0.93 (16)	5.7 ± 0.82 (59)	<0.001
**Δ24**	-1.6 ± 1.1 (58)	-1.4 ± 1.2 (69)	-1.3 ± 1.3 (40)	-1.9 ± 1.3 (15)	-1.4 ± 1.2 (54)	0.46
**Δ52**	-2.0 ± 1.3 (28)	-1.3 ± 1.5 (27)	-1.7 ± 1.4 (11)	-2.7 (1)	-2.1 ± 1.2 (8)	0.30
**JSN**						
**BL**	16.0 ± 22.0 (79)	17.8 ± 19.8 (105)	15.6 ± 24.81 (54)	13 ± 17.4 (16)	19.8 ± 34.8 (60)	0.81
**Δ24**	-0.01 ± 0.65 (71)	0.12 ± 0.7 (87)	0.05 ± 0.28 (45)	0.38 ± 1.5 (16)	0.15 ± 0.57 (55)	0.30
**Δ52**	0.32 ± 0.93 (29)	0.72 ± 1.7 (30)	0.04 ± 0.13 (14)	0 ± 0 (2)	0.18 ± 0.49 (8)	0.42
**ERN**						
**BL**	18.9 ± 21.7 (79)	20.5 ± 23.3 (105)	16.6 ± 21.0 (54)	11.3 ± 15.0 (16)	20.3 ± 37.4 (60)	0.65
**Δ24**	0.21 ± 0.69 (71)	0.47 ± 1.2 (87)	0.18 ± 0.58 (43)	0.31 ± 1.01 (16)	0.1 ± 0.81 (54)	0.15
**Δ52**	0.35 ± 0.87 (29)	1.51 ± 2.1 (30)	0.15 ± 0.43 (14)	0.5 ± 0.71 (2)	0.35 ± 0.72 (8)	0.01
**SHP**						
**BL**	34.9 ± 42.1 (79)	38.3 ± 41.3 (105)	32.2 ± 44.2 (54)	24.3 ± 31.5 (16)	40.1 ± 71.2 (60)	0.76
**Δ24**	0.2 ± 1.04 (71)	0.6 ± 1.6 (87)	0.23 ± 0.67 (43)	0.69 ± 2.5 (16)	0.28 ± 1.22 (54)	0.28
**Δ52**	0.68 ± 1.6 (29)	2.2 ± 3.4 (30)	0.18 ± 0.48 (14)	0.5 ± 0.71 (2)	0.52 ± 1.2 (8)	0.03

The absolute radiographic scores (ERN, JSN and SHP) did not differ significantly at BL between the clusters. At 52 weeks however, ΔSHP was significantly different (p = 0.03). This was driven by ΔERN which was also significantly different between the clusters (p = 0.01), with cluster B having the highest change in erosion (ΔERN = 1.51 ± 2.1). Tukey post-hoc analysis reveals ΔERN was significantly higher in B at 52 weeks than A (p<0.05) and C (p<0.05). JSN did not differ between the clusters at baseline, nor ΔJSN at week 24 or week 52.

There was a significant difference in DAS28 at baseline (p<0.001) between all clusters. Tukey post-hoc analysis shows that at baseline, DAS28 was significantly different between cluster pairs A-E, B-C and B-E. The ΔDAS28 values at week 24 and 52 however was insignificant between the clusters (Table 3).

Age, BMI, duration of the disease, number of previous DMARDs, HAQ were statistically insignificantly different between the RA placebo patients of all the clusters identified.

## Discussion

This study was a retrospective analysis of phase III clinical studies, exploring the hypothesis that biomarkers reflecting tissue metabolism may identify previously unrecognised RA endotypes. We used biomarker data from two RA clinical studies, enriched with two OA studies as a non-inflammatory arthritic population. Through clustering analysis we attempted to identify endotypes of RA with distinct profiles of tissue specific turnover, and identify associations between these endotypes and indicators of clinical outcomes. The biomarkers used are measures of tissue inflammation or turnover, mainly metabolites of collagen degradation and formation. As collagens are the main constituents of connective and calcified tissue, these biomarkers reflect the active molecular processes in disease affected tissue[33]. Type I and type III collagen are found in the connective tissue around the joint, as well as other affected connective tissues of the body, while type II collagen is present mainly in cartilage.

We found five distinct putative endotypes (i.e. clusters), associated with different biomarker levels. Clusters A and B both had high levels of connective tissue and bone turnover compared to other groups, somewhat typical of RA[7,34,35]. Conversely, clusters D and E had lower levels of the connective tissue markers. Whilst this is to be expected of OA patients, RA patients in these clusters are more unusual. These RA patients showed no significant difference in DAS28 compared to other clusters, which could indicate a subset of RA patients that have low connective tissue turnover in spite of high disease activity.

Both clusters A and B exhibit high levels of connective tissue markers and high rates of bone formation and resorption, but differ in macrophage activity (VICM) and CRP metabolism (CRPM). Interestingly, placebo patients in these clusters differ significantly in the rate of radiographic progression.

Recent studies by Gu et al. showed the role macrophages play in inflammatory bone diseases and the effect they have on bone formation and degradation. These studies record that macrophage activity plays a part in bone tissue turnover[36].

Differences between diverse inflammatory and arthritic endotypes have previously been reported by other groups[37–39]. Dennis et al. who investigated synovial biomarker traits of certain endotypes in relation to drug response. They found four potential endotypes); i) lymphoid (B and plasma cell dominant), ii) a myeloid (macrophage dominant), iii) a fibroid, and iv) a low inflammatory phenotype[37]. This may align well with the connective tissue turnover endotypes we propose in this paper, where cluster A and B are characterised by high tissue turnover driven by the MMP activity which are expressed by immune cells such as macrophages[40]. Similarly, we also found differences in the macrophage marker VICM, separating the two high inflammatory endotypes, clusters A and B.

It is also important to note that whilst disease activity (DAS28) significantly differed across the groups, the disease activity difference between groups A and B (inflammatory endotypes) was insignificant, while cluster B exhibited much faster structural progression. This finding is consistent with claims that measurement of disease activity alone it is not sufficient to identify fast progressing RA patients who may require more a more aggressive therapeutic strategy[41].

Presently, there are few positive examples of endotyping in the field of RA, other than sero-positivity, and anti–cyclic citrullinated peptide, often used to classify disease. The identification of 31 genetic risk loci in seropositive RA, including HLA–DRB1, and the group of alleles referred to as the shared epitope, demonstrate that RA has a strong but complex genetic aetiology. As a result, a genetic approach to the identification of biomarkers of response has been pursued with both candidate and genome-wide level approaches[42,43]. While associations that may ultimately lead to a greater insight into the molecular aetiology of RA have been established, the predictive capability so far has been insufficient to have meaningful clinical application. Indeed, Wang et al.[44] reported that each polymorphism identified in a genome-wide association study accounted for 2% of the variance observed with a change in Disease Activity Score in 28 joints (DAS28) response to tocilizumab, while no significant association of polymorphisms of the target IL-6 receptor and its cognate ligand IL-6 was associated with change in DAS28 response, following correction for multiple testing. Baseline serum or RNA measurement of IL-6 or IL-6 receptor levels also provided little discrimination between responders and non-responders, indicating that the prevalence of the target receptor or it’s cognate ligand was not clinically deployable to identify those patients most likely to benefit from tocilizumab[43]. There also have been attempts to utilise gene expression analysis to identify biomarkers of biologic endotypes with superior response to treatment[45]. For example, Kim et al. showed the importance of a differentially expressed gene, G0S2, in the response to anti-TNF therapy, in multiple cohorts.

The findings in this study support previous proposals that RA patients have differing underlying molecular aetiologies, and encourages further investigation into developing tools to advance patient stratification. The prospect of identifying robust endotype profiles representing distinct disease driving pathways and networks is tantalising. Such findings have implications for the development of new precision medicines, benefiting patients, physicians and payers[46]. If our endotypes are validated they may allow for superior treatment regime selection for the individual patients[47]; e.g. the treatment of patients with high bone remodelling, but moderate tissue inflammation might be focused on rebalancing the bone remodelling balance. Sivadas et al. show how whole genome sequencing has been used to identify genes involved in drug metabolism and identification of target genes[48]. In a review by Orr et al. they describe the advances in the use of synovial tissue for patient stratification and identification of novel targets and biomarkers[49]. Such approached could be coupled with a serological approach in order to improve understanding of drug metabolism and patient strata.

The strengths of this study were the large number of participants at baseline, along with robust tissue specific markers allowing for an accurate profiling and clustering of arthritic patients’ disease pathology. Due to the nature of clinical trials, a high placebo dropout rate may affect the comparison of clinical endpoints. Patients enrolled in the trials had been exposed to prior therapy including DMARDs and NSAIDs. As information regarding specific prior treatments and ACPA positivity was not recorded or made available to us, we were not able to correct for these possible confounders. In contrast to LITHE, which was a 52 week placebo-controlled study, OSKIRA was a 24 week placebo-controlled study. Hence radiographic endpoint for OSKIRA were only available up to 24 weeks. For this reason, in order to reap any practical gain from this investigation, these findings must be validated in larger cohorts with more consistent follow up measurements to show any true predictive ability of these endotypes.

## Conclusion

In conclusion this paper shows how the clustering of RA patients using tissue specific markers allows for the identification of disease endotypes, which may have use in precision medicine after continued research.
